# Factors affecting patients on antiretroviral therapy lost to follow-up in Asunafo South District of Ahafo Region, Ghana: a cross-sectional study

**DOI:** 10.1136/bmjph-2024-000944

**Published:** 2024-10-18

**Authors:** Robert Kogi, Theresa Krah, Emmanuel Asampong, Edward Mberu Kamau

**Affiliations:** 1 Health Information Unit, Asunafo South District Health Directorate, Ghana Health Service, Kukuom, Ahafo Region, Ghana; 2 Asunafo North District Health Directorate, Ghana Health Service, Goaso, Ahafo Region, Ghana; 3 School of Public Health, Department of Social and Behavioural Sciences, University of Ghana, Legon, Ghana; 4 World Health Organization Regional Office for Africa, Nairobi, Kenya

**Keywords:** Public Health, Cross-Sectional Studies, Disease Notification, HIV, Sociodemographic Factors

## Abstract

**Introduction:**

Despite the increased and effective programme coverage for antiretroviral therapy (ART), a considerable proportion of individuals receiving ART discontinue medication at different stages of their treatment pathway. In sub-Saharan Africa, approximately half of individuals who test positive for HIV are lost to follow-up (LTFU). This study was set out to answer the following question ‘What are the factors that affect patients on ART loss to follow-up in Asunafo South District of Ghana?’.

**Methods:**

Cross-sectional study design with systematic random sampling was employed to select 620 HIV patients on ART. Stata V.17.0 was used to analyse the data. A cox-proportional hazard regression was fitted in order to determine the predictor variables. Variables for the multivariable regression model were chosen by entering the outcome variable (LTFU) and explanatory variables into the model. Finally, the association between the explanatory and outcome factors was determined using the adjusted HRs and their associated 95% CI was considered.

**Results:**

A total of 600 respondents were used for the final analysis after data cleaning. Patients who began ART at age 41 years or older had a significantly lower chance of being LTFU than those who began ART at age 35 or less (adjusted HR (aHR)=0.34, 95% CI 0.13 to 0.84). Furthermore, patients who started ART with a primary education had 1.68-fold increased risk of LTFU compared with patients with no education (aHR=1.68; 95% CI 0.83 to 3.43). In addition, patients in rural locations had a 2.65-fold higher likelihood of being LTFU than patients in urban areas (aHR=2.65, 95% CI 1.29 to 5.44). The main reasons for missing ART appointments among patients included walking long distance to clinic, cost of transportation, fear of scolding from clinic staff, stigma and erratic supply of antiretrovirals.

**Conclusion:**

All clinicians should consider the risk factors that have been identified when providing ART services and counselling.

**WHAT IS ALREADY KNOWN ON THIS TOPIC**

- The existing knowledge on the topic is that despite increased and effective programme coverage for antiretroviral therapy (ART), a substantial proportion of individuals discontinue medication during various stages of treatment, particularly in sub-Saharan Africa. This study was carried out to determine the factors affecting individuals on ART loss to follow-up.

**WHAT THIS STUDY ADDS**

- As a result of this study, we now know that initiating ART at age 41 or older, having no formal employment, residing in urban areas and possessing a higher education level are associated with a significantly lower risk of lost to follow-up among HIV patients, and addressing factors such as long-distance travel, transportation costs, fear of stigma and antiretroviral supply issues are crucial in reducing patient attrition from ART.

**HOW THIS STUDY MIGHT AFFECT RESEARCH, PRACTICE OR POLICY**

- The findings suggest that older patients, those with higher education, and those in urban areas are more likely to adhere to ART. This calls for further investigation into the psychosocial, economic or health-related factors that may influence adherence among older patients. Such studies could also explore how to replicate this adherence success in younger populations. Clinicians should emphasise on personalised adherence strategies based on age, education level and geographic location in their practice. Training healthcare workers to be empathetic and culturally sensitive could help reduce patient fears and encourage continuous engagement with ART services. Policies that promote health literacy, especially among individuals with primary or no education, could help mitigate the risks associated with low education levels.

## Introduction

 The WHO has observed that HIV remains a global public health concern.^1^ Even though HIV infection cannot be cured, persons living with the virus can now live long and healthy due to improved access to HIV prevention, diagnosis, treatment and care.^2^ Regardless of clinical status or CD4 cell count, all individuals living with HIV, including children, adolescents, adults and pregnant and lactating women get lifelong antiretroviral therapy (ART).^3^ There were 342 307 estimated people living with HIV (PLHIV) in Ghana as of 2019, with a total estimated 20 068 new infections.^4^ Moreover, the estimated ART coverage among adults (15+ years) was approximately 46.6%.^4^

The WHO’s ‘treat all’ policy, which was previously used as a cut-off point to begin treatment, was accepted by the Ghanaian government.^5^ As a result, efforts to maintain the long-term effects of ART and lower the risk of new HIV infections are receiving more attention in Ghana.^6^ However, in sub-Saharan Africa patients typically have low retention rates,^7^ leading to loss to follow-up (LTFU).

The antiretroviral (ARV) coverage in Ghana is estimated to be at 45.0% among PLHIV.^8^ The immunological benefits of ARV medication are negatively impacted by LTFU, which also increases hospitalisations, morbidity and death from AIDS among HIV patients.^9^ Additionally, it was discovered that patients in Lagos, Nigeria, provided false contact details out of a refusal to accept HIV-positive results and to enrol in treatment, as well as a fear of stigma associated with the virus.^10^ In Ghana, not much research has been conducted to investigate the precise causes of HIV patient loss from ART, particularly in the Asunafo South District of the Ahafo Region. In the Asunafo South District of the Ahafo Region, report on the District Health Management System (DHIMS2) for 2020 and 2021 showed that out of 3090 and 4017 HIV patients who were on ART, some 1593 and 1564 patients, respectively, have stopped treatment due to LTFU.^11^ Knowing why people stop taking ARV medication (ART) is critical because maintaining patients on ART and ensuring adherence to therapy are critical components in achieving successful long-term outcomes. Therefore, in order to help patients on ART stay on treatment, we carried out this study to answer the question ‘What are the factors that affect patients on ART loss to follow-up in Asunafo South District of Ghana?’.

### Conceptual framework

This study sought to construct an overarching conceptual model to guide in the understanding of HIV patient LTFU in the Asunafo South District. The Andersen and Newman Behavioural Model for health service utilisation was used. The Andersen Newman Framework^12^ posits that an individual’s access to and use of healthcare is a function of three main factors: (1) predisposing factors (socio-cultural characteristics of individuals that exist prior to their illness); (2) enabling resources (the logistical aspects of obtaining care, which can include personal, family and community resources); and (3) need factors (the most immediate cause of healthcare use from problems that generate the need for care).

This framework was used because, it has both predictive and explanatory capabilities and provides insight on how to maintain, or in some cases improve, the health status of the populations under study. Additionally, the grouping of factors under study as predisposing characteristics, enabling resources and need factors can guide the development of appropriate interventions relevant to the study problem. While many predisposing characteristics such as age may be difficult to alter, to change utilisation, certain enabling resources such as type and location of care and need factors, particularly those relating to perceived needs for care, may be easier to address.^12^ Moreover, the framework allows for the consideration of broad environmental factors. Although we may not be able to measure the direct role of ART policies and tracing guidelines in this research, the framework was intended to help to identify mutable factors under study that could be affected through key policy and programme changes to reduce losses to follow-up in the district. In addition, this framework has also been used as a behavioural model for vulnerable populations, by specifically placing greater emphasis on environmental enabling resources and need factors.^13^

**Figure 1:**
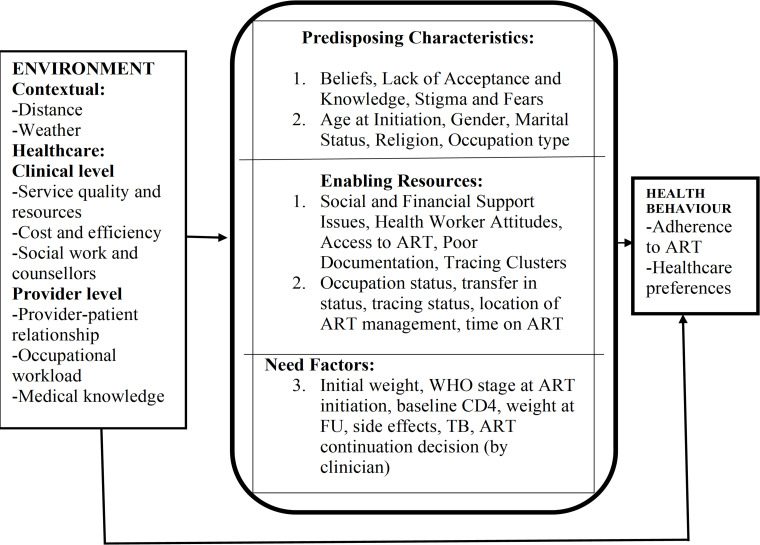
Conceptual framework of potential factors associated with becoming lost to follow-up from antiretroviral therapy (ART).

Primarily, this study was interested in identifying how various factors influence health behaviours regarding LTFU. Though they are difficult to measure directly, we recognise the importance of more distal acting environmental factors and their potential impact on health behaviours and outcomes. Hence, this conceptual model represented in figure 1 was pertinent to the research objectives of this study.

## Materials and method

### Study design

A facility-based cross-sectional study was used in this study. The data collection was conducted using the facilities’ records and tracing respondents for interview.

### Study location/area

The study was conducted in the Asunafo South District in Ahafo Region of Ghana. According to the 2020 population and housing census, the district has a total population of 93 619 with a total number of children 0–11 months and women in reproductive age being 3668 and 22 006, respectively.^14^

In terms of health service delivery, Asunafo South District is divided into 4 sub-districts; Kukuom, Sankore, Kwapong and Abuom with 20 health facilities of which 12 are functional Community-based Health Planning and Services (CHPS) distributed throughout the entire sub-districts. The district has two HIV clinics rendering ART services (Asunafo South District Hospital and Sankore Health Centre). Approximately 4017 clients were actively receiving ART by the end of 2021, and the HIV clinics are open 5 days a week. Patients from all backgrounds and communities inside and outside the district are served by the clinics.

### Variables

The outcome variable in this study was LTFU from ART services, whereas the sociodemographic variables included in the analysis were sex, age, education level, marital status, occupation status, and place of residence, the quantity of tablets taken daily, the level of adherence and adverse drug reactions were all regarded as independent variables.

### Study population

The study population included individuals who were on ART as at the end of 2021 or were no longer in care and have been LTFU. Complete location information (10-digit phone number) in the HIV testing and counselling registration was the inclusion requirement for tracing. A minimum of 18 years of age and prior experience with ART as a patient were prerequisites for inclusion.

### Sample size

The Cochran formula was used to determine the study’s sample size.^15^ The standard normal (Z) value corresponding to the 95% CI a precision level of ±5%, and an expected retention level of 72% were the parameters employed in this sample size estimation.^11^

$A\;sample\;size\;(n)=\frac{{\left(1.96\right)}^{2}\times 0.72\times \left(1-0.72\right)}{{\left(0.05\right)}^{2}}=n=310$

In order to account for clustering in LTFU at the health facility level, the sample size calculated by this method (n=310) is inflated by a design effect of two, resulting in a total sample size of 620.^16^

### Sampling method

Systematic random sampling was used to select HIV patients on ART and those LTFU. The first patient in this systematic sampling was chosen at random, and subsequent patients were then drawn from the clinics database (registers) in accordance with a sample interval defined as 5581/620 (ie, every ninth patient in each ART clinic’s register) until the targeted sample size was reached. The sample size was achieved by using the clinics register as a sampling frame.

### Data Collection Techniques/Methods & Tools

Five data collection assistants with expertise in gathering data for public health research were contracted and given training on how to collect the data. First, eligible HIV patients on ART and those on ART who stopped responding to follow-up calls were contacted to explain the objectives of the study and set up appointments for in-person structured interviews or phone interviews for those who could not be reached but were willing to take part in the study. It took at least three times to make phone calls on several days and at various times of the day to conclude that the tracing was unsuccessful.

The data was gathered using a questionnaire that was created on the KoboCollect platform. This reduced the amount of time that respondents had direct contact with the questionnaires and prevented the possible spread of COVID-19. A standardised and pretested questionnaire was used to gather the data; it was created in English and, for those who could not understand it, was translated into Twi by data collection assistants.

Data collection and patient tracing were carried out in August and September 2022. Each interview lasted from 30 to 60 min.

### Quality control

The data collection process was carefully undertaken to ensure the quality of the data, and we ensured that data collectors who were selected had prior data collection experience. Prior to data collection, a pretest was administered to 10 ART patients in the Asunafo North Municipal on populations that were similar. Throughout the whole data collection period, the investigators conducted intensive supervision daily. Before the data collection period began, the investigators examined the data collection tool to ensure its dependability. We also cross-checked the data at the end of each day to ensure it was accurate, consistent and complete. As a result, a corrective discussion was held with each data collector. During discussion periods, different views were considered to reduce errors and corrective measures were implemented. After that, the data were coded, entered the computer and examined for consistency and completeness. We used the checklist for strengthening the reporting of observational studies in epidemiology guidelines in writing this manuscript (online supplemental material 1).

### Data analysis

Excel was used to extract and verify the completeness of the data from the KoboCollect data collection application. After that, it was exported to Stata V.17.0 for processing, cleaning, editing and analysis. Each study respondent’s results were classified as either being LTFU or not LTFU. Additionally, a covariate regression model was applied in order to determine the predictor variables.

Frequency distributions or percentages were used to describe the categorical data that was taken from HIV-positive clients. In terms of bivariate analysis, variables for the multivariable covariate regression model were chosen by entering the outcome variable and explanatory variables into the covariate regression model. Thus, the multivariable covariate regression model was fitted to the variables in the bivariable analysis with p values ≤0.25. Finally, the existence of a meaningful association between the explanatory and outcome factors was determined using adjusted HR and its associated 95% CI. Significant variables were those with p values less than 0.05. The results obtained in using this section are provided as online supplemental material 2.

### Statistical methods

The statistical methods which were used in this research included descriptive statistics, covariate regression model, univariate, bivariate and multivariate analyses. The risk of LTFU was considered from the first refill period of the patient after they were put on ART. Patients who were known to have died or known to have been transferred out while still in active care were excluded from the study.

### Patient and public involvement

Participants were contacted to provide feedback on the location of interviews and the length of the survey, ensuring the process was convenient for them and respected their time. Patients were also indirectly involved in the conduct of the study through their suggestions on how to create a non-judgmental and supportive environment during data collection. The respondents were also made aware that the findings from this study would be published in a scientific journal. They were also informed that the finding from the study would be shared with the District Health Directorate and management of the various ART sites in the Asunafo South District.

## Results and discussion

## Results

### Description of the study respondents

The results in table 1 showed that majority of the respondents in this study were at least 46 years of age, 197 (32.8%), followed by respondents who were 31–35 years old, 125 (20.8%). The population studied has a median age of 36 years, which ranged from 30 to 52 years. The IQR of 22 years indicated a relatively broad spread of ages around the median, suggesting moderate age variability within this population. Majority of the respondents were females, 344 (57.3%) and a greater proportion of the respondents attained junior and or senior high school education, 372 (62.0%). A greater number of the respondents were Akans, 429 (71.5%). In addition, majority of the respondents were Christians, 526 (87.7%). Respondents who were married at the time of the conducting this study formed the greater majority of 231 (38.5%), followed by those who were single, 164 (27.3%). Also, most of the respondents who participated this study were farmers, 279 (46.5%), while most of them indicated they were urban dwellers, 459 (76.5%).

**Table 1:** Sociodemographic characteristics of respondents at the Asunafo South District

Variable	Frequency (n=600)	Percentage (100%)
Age (years)		
≤25	54	9.00
26–30	101	16.83
31–35	125	20.83
36–40	71	11.83
41–45	52	8.67
≥46	197	32.83
Median age	36	
Lower quartile	30	
Upper quartile	52	
IQR	22	
Sex		
Female	344	57.33
Male	256	42.67
Highest level of formal education		
None	96	16.00
Primary	101	16.83
JHS/SHS	372	62.00
Vocational	16	2.67
Tertiary	15	2.50
Ethnicity		
Akan	429	71.50
Dagomba/Dagaabas/Gonja	90	15.00
Ewe	30	5.00
Fante	37	6.17
Krobo	14	2.33
Religion		
Christian	526	87.67
Muslim	74	12.33
Marital status		
Cohabiting	59	9.83
Divorced/widowed	146	24.33
Married	231	38.5
Single	164	27.33
Occupation		
Unemployed	96	16
Artisan	28	4.67
Trading	84	14
Dressmaker	23	3.83
Farmer	279	46.5
Government worker	15	2.5
Other	75	12.5
Locality		
Rural	141	23.5
Urban	459	76.5

### Use of ARV among study respondents

From table 2, it is shown that majority of the respondents were on ARVs, 564 (94.0%) at the time of conducting this survey. Among those respondents who were on ARVs, a greater proportion of them were taking first (first) dose (first line), 564 (94.0%). It is further shown that majority of the respondents who were on ARVs like zidovudine (AZT), lamivudine (3TC), tenofovir disoproxil fumarate (TDF), emtricitabine (FTC) and abacavir (ABC), indicated they regularly took their drugs, 534 (94.7%). For the respondents who were on ARVs, a greater number of them said they took them from the clinic, 549 (97.3%). Finally, though a majority (68.6%) of the respondents indicated they never missed their ARVs, some 22.5% and 5.0%, respectively, said the last time they missed their ARVs were 1–4 weeks and more than 3 months ago prior to the conduct of this survey.

**Table 2:** Use of antiretroviral among ART patients

Variable	Frequency (n=564)	Percentage (100%)
On ARV		
No	36	6.00
Yes	564	94.00
Number of doses taken a day		
One dose (first line)	557	98.76
Two doses (second line)	7	1.24
Still on ART		
Yes always	534	94.68
Yes sometimes	30	5.32
Source of ARVs		
Clinic (s)	549	97.34
Pharmacy	15	2.66
Last time respondents missed ARVs		
1–4 weeks	127	22.52
Within the past week	22	3.90
>3 months	28	4.96
Never missed ARVs	387	68.62

ARTantiretroviral therapyARVantiretroviral

### Reasons for discontinuing ARV medication

The results in table 3 showed that in terms of access to care, the major reasons why some respondents may discontinue care were walking long distance to clinic, 352 (58.7%) and high cost of transportation, 286 (47.7%). In terms of clinic quality, majority of the respondents discounted the assertion that staff were not nice, 537 (89.5%), were afraid to be scolded by clinic staff, 586 (97.7%), and that attending clinic did not risk disclosure of their status to community, 570 (95.0%). It is however shown in the results that many of the respondents noted that feeling healthy, 332 (55.3%) was one of their personal reasons why they may not continue with the intake of ARVs. Though majority of the respondents said they needed the ARVs, 558 (93.0%), some 42 (7.0%) indicated that they did not need it. Also, most of the respondents refuted the assertion that they were not permitted by religion or faith to continue with the intake of their ARVs, 571 (95.2%). However, a proportion of 29 respondents (4.8%) confirmed that they were not permitted by their religion or faith to continue taking ARVs.

**Table 3:** Reasons for discontinuation of care among ARV patients

Variable	Frequency (n=600)	Percentage (100%)
	Yes, n (%)	No, n (%)
Access to care		
Walk long distance to clinic	352 (58.67)	248 (41.33)
Long waiting time	28 (4.67)	572 (95.33)
Started treatment in another clinic	60 (10.00)	540 (90.00)
High cost of transportation	286 (47.67)	314 (52.33)
High cost of test	14 (2.33)	586 (97.67)
Other reasons	35 (5.83)	565 (94.17)
Clinic quality		
Staff not nice	63 (10.50)	537 (89.50)
Afraid to be scolded by clinic staff	14 (2.33)	586 (97.67)
Attending clinic can disclose my status	30 (5.00)	570 (95.00)
Personal/family and work		
Busy at workplace	7 (1.17)	593 (98.83)
Busy caring for family	14 (2.33)	586 (97.67)
A family member died	21 (3.50)	579 (96.50)
Feeling healthy	332 (55.33)	268 (44.67)
Didn’t need ARV	42 (7.00)	558 (93.00)
Treatment alternative and advice		
Religion or faith did not permit	29 (4.83)	571 (95.17)
Other treatment alternatives	7 (1.17)	593 (98.83)

ARVantiretroviral

### Predictors of patients LTFU

Table 4 depicts the unadjusted, full adjusted and final adjusted hazard rates of factors associated with LTFU.

**Table 4:** Hazard ratios of patients lost to follow-up (LTFU) being unadjusted and adjusted among 600 respondents

Variables	Total	Total LTFU	Unadjusted HR (95%CI)	P value	Adjusted HR (95% CI)	P value
Age						
≤35	155	16	Ref		Ref	
36–40	125	5	1.78 (0.01 to 2.12)	0.003	0.05 (0.27 to 0.28)	0.000
≥41	320	15	0.34 (0.18 to 0.66)	0.001	0.34 (0.13 to 0.84)	**0.020**
Highest education						
None	95	1	Ref		Ref	
Primary	101	20	6.02 (0.03 to 8.12)	0.000	1.68 (0.83 to 3.43)	**0.000**
JHS/SHS	372	12	1.13 (0.02 to 2.23)	0.240	0.44 (0.25 to 5.24)	0.265
Vocational	16	2	3.08 (0.08 to 4.51)	0.180	0.10 (0.03 to 2.80)	0.254
Tertiary	15	1	3.07 (0.05 to 5.08)	0.100	0.45 (0.34 to 1.26)	0.082
Sex						
Female	344	22	Ref			
Male	256	14	0.92 (0.47 to 1.79)	0.796	–	
Ethnicity						
Akan	429	27	Ref		Ref	
Dagomba/Dagarti/Gonja	90	2	2.55 (0.24 to 3.20)	0.111	0.17 (0.09 to 2.58)	0.095
Ewe	30	1	2.54 (0.12 to 5.34)	0.241	0.02 (0.01 to 1.05)	0.241
Fante	37	5	2.37 (1.03 to 5.41)	0.041	1.72 (0.56 to 5.24)	0.343
Krobo	14	1	2.54 (0.02 to 4.25)	0.210	0.02 (0.01 to 1.25)	0.120
Religion						
Christians	526	34	2.58 (0.52 to 3.02)	0.280	–	
Islamic	74	2	Ref			
Marital status						
Single	164	22	Ref		–	
Cohabiting	59	2	2.08 (0.05 to 2.51)	0.120		
Divorced/widowed	146	4	2.09 (0.01 to 3.52)	0.280		
Married	231	6	0.41 (0.21 to 0.81)	0.010		
Occupation						
Unemployed	96	21	Ref		Ref	
Non-government workers	489	13	0.12 (0.06 to 0.24)	0.000	0.31 (0.14 to 0.69)	**0.004**
Government workers	15	2	1.72 (0.20 to 2.32)	0.110	1.20 (0.05 to 2.13)	0.241
Location						
Urban	459	15	Ref		Ref	
Rural	141	21	4.92 (2.53 to 9.54)	0.000	2.65 (1.29 to 5.44)	**0.008**

Bold values means statistically significance.

In the final model, the risk of LTFU was shown to be considerably lower among respondents who started ART at aged 36–40 (adjusted HR (aHR)=0.05, 95% CI 0.27 to 0.28) and 41 years and above compared with those who started ART at age 35 or below (aHR=0.34, 95% CI 0.13 to 0.84). Compared with respondents without any education, those who began ART and had only completed primary school had a 1.68-fold higher chance of dropping out of the study (aHR=1.68; 95% CI 0.83 to 3.43). Comparing respondents who were unemployed to those who were employed, there was a 69% reduction in LTFU (aHR=0.31, 95% CI 0.14 to 0.69). Patients who lived in rural areas were 2.65 times more likely to get LTFU care than patients who lived in urban areas (aHR=2.65, 95% CI 1.29 to 5.44).

## Discussion

The purpose of this study was to identify the factors that affected ART patients’ failure to remain on ART at public health institutions in Asunafo South District. Of the 620 targeted sample size for this study, 600 patients representing about 96.8% who participated had their data fully completed and qualified for consideration in the analysis.

More than half (57.3%) of the respondents in this study were females. This result is lesser than what was reported in another study in Nigeria that about 70.5% of the respondents on ART were females.^17^ This study’s findings reveal that, despite most respondents being on ART, a small percentage of them (6.0%) were not taking their medications regularly in the Asunafo South District, Ghana. This indicates the presence of a gap in adherence to ARV treatment among some PLHIV. The result is in line with what was reported in Southern Ethiopia which found that 9.1% of HIV patients did not continue receiving treatment after starting it.^9^

Among the respondents on ARV treatment, a significant proportion (94.0%) were on first-line regimens, and nearly all (94.7%) reported regular adherence to their prescribed medication. Moreover, most of the respondents preferred to receive their ARVs from the clinic (97.3%), and this preference is consistent with a finding from Ghana that the majority of PLHIV preferred to acquire ART drugs from medical professionals, and the hospital was the most popular location for these prescriptions.^18^ The preference of patients receiving ARVs from the clinic and health personnel in this study indicates the importance of ensuring adequate access to these resources for PLHIV.

This study identified key factors associated with ARV treatment discontinuation among PLHIV. These include barriers to access to care such as long distance to clinics, high cost of transportation and long waiting times. We found that some respondents were concerned about the quality of care, including concern of being badly treated by health workers (10.5%), others were also afraid of scolding from clinic staff (2.3%), while some patients said attending the clinic risked disclosure of their status to the community (5.0%). Other research expressed a similar concern, stating that maintaining a good rapport with the clinic staff was essential to keeping patients there.^19 20^ This current study’s findings also supported what was reported in southern Mozambique, that the most frequently reported barriers to patients LTFU were the fear of bad treatment from health personnel.^21^

Furthermore, the results of this study showed that a little more than half of the respondents (55.3%) stated that they were not ready to continue taking ARVs since they were feeling well, and almost 7.0% of them felt they did not need the ARVs at all, because they were feeling healthy. This inconsistent use of ARVs can increase the viral load in individuals, leading to a higher risk of HIV transmission to others.^22^ This finding corroborated with what was found in southern Mozambique, that one of the most frequently reported barriers to patients LTFU was the perception of being in good health.^21^

Also, although most of the respondents refuted the assertion that they were not permitted by religion or faith to continue with the intake of their ARVs in this study, it was found that a proportion of about 4.8% confirmed that they were not permitted by their religion or faith to continue taking their ARVs. Other respondents, seven (1.2%) have ever sought HIV treatment from alternate sources. A similar finding was reported that patients believed that God could ‘cure HIV’ and sought their care from different places instead of the hospital.^23^ This could be affecting clients’ readiness and adherence to the ARVs medications. A similar finding was reported in Kumasi-Ghana, that the study respondents believed alternative or herbal remedies could cure their HIV infections.^24^

### Predictors of clients LTFU

Respondents who started ART at age 36–40 years as well as those who were 41 years old and above had a much lower risk of long-term follow-up than patients who started ART at age 35 or below. This difference in risk was statistically significant. This result suggested that, in comparison to older age groups, younger patients were more likely to be LTFU from ART. One possible reason could be that younger patients are more likely than older patients to be mobile and to fear stigma associated with HIV.^25^ This may also be related to patients’ immaturity in their ability to think critically and the unique difficulties that come with being younger than older patients. This finding agreed with what was reported that younger age could result in LTFU and that age less than 35 years was a predictor of LTFU from ART by.^9 26^

Also, patients who started ART with a primary education level had a 1.68-fold increased risk of LTFU compared with those without any education. This finding could be attributed to the fact that patients with primary education may have a basic understanding of health and disease, which could lead to a false sense of confidence in managing their condition. They might believe that they can manage their treatment without strict adherence to follow-up appointments or may misunderstand the importance of continuous monitoring and medication adherence. Also, education levels can influence how patients perceive stigma and seek social support. Patients with primary education might be more sensitive to stigma but less equipped to manage it compared with those with no formal education, who may have developed stronger community ties or alternative support networks. This stigma can deter them from attending follow-up appointments or engaging with healthcare services. This finding disagreed with the findings reported in another study conducted in South Sudan which reported that that LTFU was high in patients who had low level of education.^26^ Furthermore, a little above two-thirds of patients who were not government workers had a significantly lower likelihood of becoming LTFU than patients who were unemployed. This implied that patients who were employed in one way or the other may be a bit financially stable and may perceived seeking care at different places and sources as compared with the unemployed who may lack money, and had no option than to ensure regular follow-up for their ART. This supported what was reported in North-western Ethiopia that lack of employment was a strong predictor of not receiving ART.^27^ In this present study, we also discovered that patients were 2.65 times more likely to be LTFU in rural areas than they were in urban settings. This may be the result of issues with accessibility or distance from treatment facilities, transportation expenses, a lack of knowledge about the advantages and risks of treatment adherence, and societal stigma associated with HIV and AIDS among patients from rural areas.

The study faces several methodological limitations that were considered during the interpretation of its findings. The study did not consider variables such as socioeconomic status or health conditions at the time of ART initiation, which could have influenced the observed associations between age, education and geographic location with the likelihood of being LTFU. Measurement bias was another issue, especially as the study relied on self-reported data for reasons of missed appointments or educational background, which can be subject to inaccuracies. Moreover, geographical and cultural variability limited the applicability of the findings to other regions, as rural and urban challenges, as well as stigma, can vary significantly across different cultural contexts.

Furthermore, our study was constrained by the fact that, in order to account for potential bias, we did not compare patients’ missing information to those who did not, on important characteristics associated with LTFU. Also, a few factors that may have been overlooked, such as having a caregiver, having a viral load, having a mental disorder and body mass index, could also be predictive factors for LTFU.

## Conclusion

This study contributes significantly to the existing body of knowledge on HIV treatment, shedding new light on key factors influencing treatment outcomes in Ghana. First, compared with patients who started ART at a younger age, this study revealed that patients who were above 35 years old had a considerably decreased risk of LTFU. This finding provides crucial insight, highlighting the need to prioritise younger patients in ART treatment to improve adherence. Second, the finding that patients with only primary education were more likely to experience LTFU than patients with no education points to the need of health education programmes to increase low-education patients’ adherence to ART.

Furthermore, it was demonstrated that patients who did not work for the government had a lower likelihood of LTFU than the unemployed. A noteworthy finding that emphasises the need to address the difficulties rural patients encounter in getting and sticking to ART is that they were most likely to terminate their ART treatment than patients who lived in urban areas. Finally, this study has identified various reasons for missing ART appointments, such as the cost of transportation, stigma and perceived side effects of the drugs, provided valuable insights into the factors that influenced patients LTFU from ART.

Therefore, all physicians should consider the risk factors that have been identified in this study when providing ART services and counselling.

### Policy recommendations

- Ghana AIDS Commission in collaboration with Ghana Health Service should develop strategies to include interventions to address the concerns of patients about the quality of care, fear of stigmatisation and other barriers to access care.
- Ghana AIDS Commission in collaboration Ghana Health Service should train health personnel to provide quality care and support and providing psychosocial support services to address the fears and concerns of patients.
- Ghana AIDS Commission, Ghana Health Service and other stakeholders should develop targeted interventions to address the needs of younger patients and patients with lower levels of education who are at a higher risk of LTFU. Strategies could include improving education and awareness about HIV and ART, providing support for transportation and other related costs, and improving access to care for patients who are unemployed or have low incomes.

## supplementary material

10.1136/bmjph-2024-000944 online supplemental file 1

10.1136/bmjph-2024-000944 online supplemental file 2

## Data Availability

The data for this study is available upon official request from the corresponding author through email: robertkogi87@gmail.com
