# Supplementary material for: Factors affecting patients on antiretroviral therapy lost to follow-up in Asunafo South District of Ahafo Region, Ghana: a cross-sectional study
Source: BMJ Public Health. 2024 Oct 18;2(2):e000944. doi: 10.1136/bmjph-2024-000944 (PMC11816434; doi:10.1136/bmjph-2024-000944)
Supplement: online supplemental file 2 [file bmjph-2-2-s002.pdf]

Table 1: Socio-demographic Characteristics of Respondents at the Asunafo South District

Variable	Frequency (n=600)	Percentage (100%)
**Age (Years)**		
≤25	54	9.00
26-30	101	16.83
31-35	125	20.83
36-40	71	11.83
41-45	52	8.67
≥46	197	32.83
Median age	36	
Lower quartile	30	
Upper quartile	52	
Interquartile Range	22	
**Sex**		
Female	344	57.33
Male	256	42.67
**Highest level of formal education**		
None	96	16.00
Primary	101	16.83
JHS/SHS	372	62.00
Vocational	16	2.67
Tertiary	15	2.50
**Ethnicity**		
Akan	429	71.50
Dagomba/Dagaabas/Gonja	90	15.00
Ewe	30	5.00
Fante	37	6.17
Krobo	14	2.33
**Religion**		
Christian	526	87.67
Muslim	74	12.33
**Marital Status**		
Co-habiting	59	9.83
Divorced/Widowed	146	24.33
Married	231	38.5
Single	164	27.33
**Occupation**		
Unemployed	96	16
Artisan	28	4.67
Trading	84	14
Dressmaker	23	3.83
Farmer	279	46.5
Government Worker	15	2.5
Other	75	12.5
**Locality**		
Rural	141	23.5

Urban	459	76.5

Table 2: Use of antiretroviral among ART patients

Variable	Frequency (n=564)	Percentage (100%)
**On ARV**		
No	36	6.00
Yes	564	94.00
**Number of doses taken a day**		
1 dose (first line)	557	98.76
2 doses (second line)	7	1.24
**Still on ART**		
Yes always	534	94.68
Yes sometimes	30	5.32
**Source of ARVs**		
Clinic (s)	549	97.34
Pharmacy	15	2.66
**Last time respondents missed ARVs**		
1-4 weeks	127	22.52
Within the past week	22	3.90
>3 months	28	4.96
Never missed ARVs	387	68.62

Table 3: Reasons for discontinuation of care among ARV patients

Variable	Frequency (n=600)	Percentage (100%)
	Yes, n (%)	No, n (%)
**Access to care**		
Walk long distance to clinic	352 (58.67)	248 (41.33)
Long waiting time	28 (4.67)	572 (95.33)
Started treatment in another clinic	60 (10.00)	540 (90.00)
High cost of transportation	286 (47.67)	314 (52.33)
High cost of test	14 (2.33)	586 (97.67)
Other reasons	35 (5.83)	565 (94.17)
**Clinic quality**		
Staff not nice	63 (10.50)	537 (89.50)
Afraid to be scolded by clinic staff	14 (2.33)	586 (97.67)
Attending clinic can disclose my status	30 (5.00)	570 (95.00)
**Personal/ family and work**		
Busy at workplace	7 (1.17)	593 (98.83)
Busy caring for family	14 (2.33)	586 (97.67)
A family member died	21 (3.50)	579 (96.50)
Feeling healthy	332 (55.33)	268 (44.67)
Didn’t need ARV	42 (7.00)	558 (93.00)
**Treatment alternative and advice**		

religion or faith did not permit	29 (4.83)	571 (95.17)
Other treatment alternatives	7 (1.17)	593 (98.83)

Table 4: Hazard ratios of patients lost to follow-up being unadjusted and adjusted among 600 respondents

Variables	Total	Total LTFU	Unadjusted HR (95%CI)	p-value	Adjusted aHR (95% CI)	p-value
Age						
≤35	155	16	Ref		Ref	
36-40	125	5	1.78(0.01-2.12)	0.003	0.05(0.27-0.28)	0.000
≥41	320	15	0.34(0.18-0.66)	0.001	0.34(0.13-0.84)	**0.020**
**Highest education**						
None	95	1	Ref		Ref	
Primary	101	20	6.02(0.03-8.12)	0.000	1.68(0.83-3.43)	**0.000**
JHS/SHS	372	12	1.13(0.02-2.23)	0.240	0.44(0.25-5.24)	0.265
Vocational	16	2	3.08(0.08-4.51)	0.180	0.10(0.03-2.80)	0.254
Tertiary	15	1	3.07(0.05-5.08)	0.100	0.45(0.34-1.26)	0.082
**Sex**						
Female	344	22	Ref			
Male	256	14	0.92(0.47-1.79)	0.796	-	
**Ethnicity**						
Akan	429	27	Ref		Ref	
Dagomba/Dagarti/Gonja	90	2	2.55(0.24-3.20)	0.111	0.17(0.09-2.58)	0.095
Ewe	30	1	2.54(0.12-5.34)	0.241	0.02(0.01-1.05)	0.241
Fante	37	5	2.37(1.03-5.41)	0.041	1.72(0.56-5.24)	0.343
Krobo	14	1	2.54(0.02-4.25)	0.210	0.02(0.01-1.25)	0.120
**Religion**						
Christians	526	34	2.58(0.52-3.02)	0.280	-	
Islamic	74	2	Ref			
**Marital status**						
Single	164	22	Ref		-	
Co-habiting	59	2	2.08(0.05-2.51)	0.120		
Divorced/Widowed	146	4	2.09(0.01-3.52)	0.280		
Married	231	6	0.41(0.21-0.81)	0.010		
**Occupation**						
Unemployed	96	21	Ref		Ref	
Non-Government Workers	489	13	0.12(0.06-0.24)	0.000	0.31(0.14-0.69)	**0.004**
Government Workers	15	2	1.72(0.20-2.32)	0.110	1.20(0.05-2.13)	0.241
**Location**						
Urban	459	15	Ref		Ref	
Rural	141	21	4.92(2.53-9.54)	0.000	2.65(1.29-5.44)	**0.008**

HR: Hazards Proportional Ratio
Ref: Reference group
